# Prokaryotic and Eukaryotic Fecal Microbiota in Irritable Bowel Syndrome Patients and Healthy Individuals Colonized With *Blastocystis*

**DOI:** 10.3389/fmicb.2021.713347

**Published:** 2021-09-17

**Authors:** Céline Nourrisson, Julien Scanzi, Julie Brunet, Frédéric Delbac, Michel Dapoigny, Philippe Poirier

**Affiliations:** ^1^Service de Parasitologie-Mycologie, CHU de Clermont-Ferrand, 3IHP, INSERM, Université Clermont Auvergne, Clermont-Ferrand, France; ^2^Service de Médecine Digestive et Hépatobiliaire, CHU de Clermont-Ferrand, Université Clermont Auvergne, Clermont-Ferrand, France; ^3^CNRS, Université Clermont Auvergne, Aubière, France

**Keywords:** *Blastocystis*, gut microbiota, irritable bowel syndrome, IBS-C, eukaryome, 16S/18S ribosomal RNA gene analysis

## Abstract

*Blastocystis* is the most frequently isolated protozoan from human stool. Its role in human health is still debated, and a high prevalence was reported in irritable bowel syndrome (IBS) subjects, suggesting a potential link with microbiota. In the present study, we aimed to investigate prokaryotic and eukaryotic microbiota in both IBS-C (constipated) and healthy individuals. We recruited 35 IBS-C patients and 23 healthy subjects, from which 12 and 11 carried *Blastocystis*, respectively. We performed 16S and 18S rRNA high-throughput sequencing on feces. Whereas we did not observe differences between infected and non-infected controls, several phyla were significantly modified in IBS-C patients according to the presence of *Blastocystis*. *Tenericutes* phylum and *Ruminococcaceae* family were especially increased in *Blastocystis* carriers. Furthermore, colonization with *Blastocystis* was associated with discrete changes in the microbial eukaryome, particularly among the *Fungi* taxa. Depending on the group of patients considered, the mycobiota changes do not go in the same direction and seem more deleterious in the IBS-C group. These results encourage further *in vivo* and *in vitro* investigations concerning the role of *Blastocystis* in the gut environment.

## Introduction

*Blastocystis* is the most prevalent intestinal parasite found in human worldwide, even in industrialized countries; for example, the prevalence is approximately 17% in France (El Safadi et al., 2016). Twenty-two subtypes (ST) of the parasite have been described using a 600-bp barcode sequence of the 18S rRNA encoding gene (Stensvold and Clark, 2020). Among them, ST1 to ST9 and ST12 were identified in human stools, with ST1 to ST4 being the most frequent (Alfellani et al., 2013; Ramírez et al., 2016). *Blastocystis* pathogenicity remains debated as most of the colonized people are asymptomatic. A potential link between *Blastocystis* and irritable bowel syndrome (IBS), a functional chronic disorder, was suspected in several studies on the basis of prevalence data and potential virulence factors produced by the parasite (Poirier et al., 2012; Nourrisson et al., 2014, 2016). Four symptom-based subgroups of IBS can be distinguished according to the predominant bowel habit: IBS-C for patients with constipation, IBS-D for patients with diarrhea, IBS-M for patients with alternating diarrhea and constipation, and IBS-U for patients with unsubtyped IBS.

Investigations in asymptomatic subjects carrying *Blastocystis* are required, given (i) the large number of colonized people, and (ii) questions about the infectious risks link with fecal microbiota transplantation (FMT) and its consequences on the health of recipients. Indeed, *Blastocystis*-colonized people should be excluded from donation, even if they are otherwise in good health, since the safety of transplanting the parasite has not been demonstrated (Cammarota et al., 2017).

These concerns have led to the publication of a growing number of studies on the impact of *Blastocystis* on the intestinal microbiota, focusing mainly on its prokaryotic component (reviewed in Deng et al., 2021). Many studies support that *Blastocystis* is associated with higher bacterial diversity and richness, and that the parasite could be considered as a commensal of a healthy gut environment. However, the debate remains wide open since other works identify a decrease of the abundance of beneficial bacteria such as *Bifidobacterium* sp. (Nourrisson et al., 2014). A decrease in *Bifidobacterium* and *Lactobacillus* was also observed in mice infected with ST7 (Yason et al., 2019). Moreover, Defaye et al. (2020) recently demonstrated that *Blastocystis* infection in rat is responsible for microbiota changes associated with visceral hypersensitivity. These changes included an increase in the relative abundance of *Oscillospira* and a decrease in *Clostridium*.

Works studying the eukaryotic microbiota are rarer. One study reported that *Blastocystis* colonization is associated with an increase in fungal diversity of intestinal tract (Nieves-Ramírez et al., 2018).

Facing these contradictory results and the need of additional data for microbiota modification associated with *Blastocystis* colonization, and especially interactions with eukaryotic microbiota, we conducted in the present work a prospective study in both healthy and IBS-C subjects.

## Materials and Methods

### Patient Recruitment

From January 2014 to July 2017, patients suffering from IBS-C and healthy subjects were recruited in a prospective study. Both IBS-C (fulfilling the Rome III criteria, Longstreth et al., 2006) and healthy subjects beneficiated of a medical consultation with a gastroenterologist in the Gastroenterology unit of the University Hospital of Clermont-Ferrand (France). All included subjects were men or women over the age of 18. Subjects who used antibiotics or probiotics less than 2 months before stool collection were not included in the study, as are control subjects with functional digestive disorders or other known intestinal disease. IBS symptom severity score (IBS-SSS, also known as Francis score), BMI, sex, and age were collected. This clinical study was approved by the research ethics committees of the Clermont-Ferrand Hospital (“Comité de Protection des Personnes Sud-Est VI,” France) with the reference number 2013-A00031-44.

### Stool Samples and DNA Extraction

Stool specimens from included subjects were processed in less than 4 h after emission. Approximately 200 mg of stools was mechanically ground with 0.5-mm-diameter glass beads on Tissue Lyser (Qiagen) during 3 min at 30 Hz/s. Total DNA extraction was then performed with the QIAamp^®^ DNA Stool Mini Kit (Qiagen) and eluted in a final volume of 200 μl according to the manufacturer’s recommendations. DNA extracts were stored at –80°C until the next steps.

### Detection and Subtyping of *Blastocystis* spp.

Specific quantitative PCR (qPCR) to detect and subtype *Blastocystis* was carried out using BL18SPPF1/BL18SR2PP primers (Supplementary Table 1) that target a conserved region of the SSU rRNA gene and allow discrimination between subtypes as previously described (Poirier et al., 2011). *Blastocystis* subtypes were assigned with a query coverage > 98% with exact match or identity > 98%.

### 16S rRNA and 18S rRNA High-Throughput Sequencing

Illumina high-throughput sequencing was performed by MRDNA lab (^1^ Shallowater, TX, United States) on a MiSeq (Macrogen, Inc.) following the manufacturer’s guidelines. Briefly, the variable regions V3–V5 of the bacterial 16S rRNA gene were amplified using the broad-range forward primer 515F and the reverse primer 909R (Li et al., 2016; Supplementary Table 1). Similarly, a portion of the sequence of the 18S rRNA gene was amplified using the primers 515F_Euk and 1119R previously described (Parfrey et al., 2014; Supplementary Table 1). For each target, a 28-cycle PCR (5 cycles used on PCR products) was performed with barcoded forward primers and using the HotStarTaq Plus Master Mix Kit (Qiagen, Germantown, MD, United States) under the following conditions: 94°C for 3 min, followed by 28 cycles of 94°C for 30 s, 53°C for 40 s, and 72°C for 1 min, after which a final elongation step at 72°C for 5 min was performed. After amplification, PCR products were checked in 2% agarose gel to determine the success of amplification and the relative intensity of bands. Multiple samples were pooled together (e.g., 100 samples) in equal proportions based on their molecular weight and DNA concentrations. Pooled samples were purified using calibrated Ampure XP beads. Then, Illumina sequencing was performed and DNA libraries were built according to the Illumina TruSeq DNA library preparation protocol.

### Sequencing Data Quality Control and Preprocessing

Sequence data were processed using QIIME microbiome analysis package (Quantitative Insights into Microbial Ecology QIIME, version 1.8.0^2^) (Caporaso et al., 2010).

#### 16S rRNA Gene Sequences

In summary, sequences were demultiplexed to remove barcodes and primer sequences. Sequences < 250 bp or > 600 bp or with ambiguous base calls were removed. Operational taxonomic units (OTUs) based on 97% specific 16S rRNA gene sequence identities were generated with the SILVA database (release 138^3^) using uclust, and chimeras were removed using ChimeraSlayer. Final OTUs were taxonomically classified using BLASTn against SILVA database. Data sets were filtered to exclude singletons and mitochondrion and chloroplast sequences. Following filtering, a cutoff of 650 reads per sample was applied. All 16S rRNA gene samples passed the cutoff.

#### 18S rRNA Gene Sequences

In summary, sequences were demultiplexed to remove barcodes and primer sequences. Sequences < 200 bp or > 1,200 bp or with ambiguous base calls were removed. Sequences were clustered in OTUs using an open reference strategy with the SILVA database (release 138), and chimeras were removed using ChimeraSlayer. Final OTUs were taxonomically classified using BLASTn against SILVA database. Data sets were filtered to exclude singletons and mammalian and plant sequences. Following filtering, a cutoff of 650 reads per sample was applied.

### Functional Bioinformatic Analysis of 16S rRNA Data

R package Tax4Fun^4^ was used to predict the functional capabilities of the microbial communities based on 16S rRNA data (Aßhauer et al., 2015). The OTU table generated with QIIME was imported to Tax4Fun (R version 4.0.3) and the SILVA123 database was used to predict functional capabilities. Briefly, the linear relationship between the SILVA classification and Kyoto Encyclopedia of Genes and Genomes (KEGG) database prokaryotic classification realized the prediction of the microbial community function. A heat map was built based on gene abundances of the enzymes of interest extracted from the output tables of Tax4Fun.

### Statistical Analysis

Alpha diversity, i.e., the richness of single microbial taxa within a sample, was measured using observed OTU, Chao 1, and Shannon’s and Simpson’s indexes. Observed OTU measurements were determined with QIIME using an OTU table rarefied at various depths. Boxplots showing alpha diversity were created in QIIME. Monte Carlo permutations were used to calculate the *p*-values.

Beta diversity, i.e., the variation in microbiota composition between individual samples, was assessed based on both weighted and unweighted UniFrac distances metrics between samples computed with the rarefied OTUs count table. Principal coordinates analysis (PCoA), generated in QIIME, was used to further assess and visualize beta diversity. PCoA was created in R software (4.0.3).

LefSe (LDA Effect Size) was used to investigate bacterial members that drive differences between groups (Segata et al., 2011).

For non-Gaussian data, comparisons were performed by the non-parametric Mann–Whitney *U* test (unpaired data). A *p*-value ≤ 0.05 was considered statistically significant. All statistical analyses were conducted in R software (4.0.3).

## Results

Thirty-five patients suffering from IBS-C (8 males and 27 females, sex ratio 0.30) and 23 healthy subjects (10 males and 13 females, sex ratio 0.77) were recruited (Supplementary Table 2). *Blastocystis* was detected among 12 patients of the IBS-C group (34.3%) and among 11 subjects of the control group (47.8%). In the IBS-C group, the most frequent subtype (ST) was ST4 (*n* = 4), followed by ST3 (*n* = 3), ST2 (*n* = 3 including one co-infection), ST1 (*n* = 1), ST5 (*n* = 1 co-infection), and ST7 (*n* = 1). In the control group, the ST distribution was as follows: 4 ST1, 3 ST3, 3 ST4, and 1 ST7. Body mass index (BMI) was not significantly different between the two groups (23.4 in the IBS group and 26.2 in the control group, Mann–Whitney test, *p* = 0.096) or according to *Blastocystis* carriage (IBS group: 24.8 in *Blastocystis* carriers and 23.1 in non-carriers, *p* = 1.000; control group: 24.0 in *Blastocystis* carriers and 28.7 in non-carriers, *p* = 0.141). In the IBS group, Francis score was not significantly different between carriers and non-carriers of *Blastocystis* (277 and 324, respectively, Mann–Whitney test, *p* = 0.270).

**FIGURE 1:**
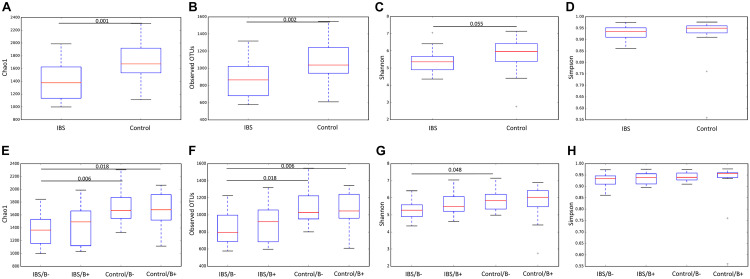
Comparison of alpha diversity indices of the fecal bacterial microbiota between controls **(E–H)** and IBS-C subjects **(A–D)** according to *Blastocystis* carriage. **(A,E)** Chao 1, **(B,F)** observed number of OTUs, **(C,G)** Shannon diversity, and **(D,H)** Simpson’s index. Significant (or close to significance) *p*-values were reported on boxplots. IBS/B-: *Blastocystis*-negative IBS patients; IBS/B + : *Blastocystis*-positive IBS patients; Control/B-: *Blastocystis*-negative control subjects; Control/B + : *Blastocystis*-positive control subjects.

We first analyzed the 16S rRNA sequence data set. Our analyses revealed a significant decrease in bacterial richness (Chao 1 and observed number of OTUs) in IBS-C compared with controls (Figures 1A,B). Diversity (Shannon function and Simpson’s index) was not significantly different between IBS-C and control groups, but there was a trend to Shannon diversity decrease, which depends more on less abundant species than Simpson’s index (Figures 1C,D). There was no significant difference of alpha diversity metrics within the control and IBS groups depending on whether *Blastocystis* was present or not (Figures 1E–H). However, bacterial richness was significantly decreased in IBS-C compared to both carriers and non-carriers in control groups. Interestingly, both bacterial richness and diversity tend to be increased in IBS-C *Blastocystis*-positive subjects compared to -negative subjects, but the difference was not significant.

**FIGURE 2:**
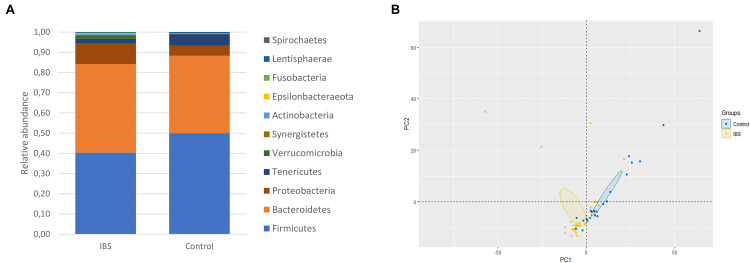
Bacterial alterations in IBS. **(A)** Comparison of the fecal bacterial microbiota composition between controls and IBS-C subjects at the phylum level. **(B)** PCoA of the unweighted UniFrac distance of control (blue plots) and IBS (yellow plots) subjects.

**FIGURE 3:**
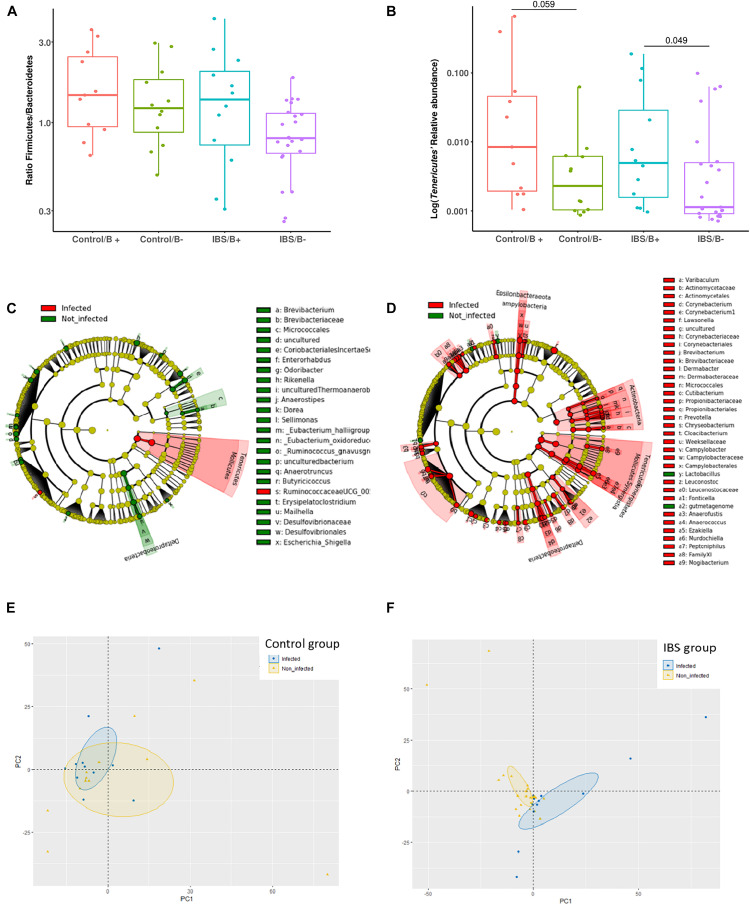
Investigations about bacterial phyla/members that drive differences in each group of patients according to *Blastocystis* carriage. **(A,B)** Analysis at the phyla level: **(A)** *Firmicutes/Bacteroidetes* ratio in controls and IBS according to *Blastocystis* carriage. **(B)** Relative abundance of *Tenericutes*. Statistical significance was determined by Mann–Whitney test. Significant (or close to significance) *p*-values were reported on boxplots. IBS/B-: *Blastocystis*-negative IBS patients; IBS/B + : *Blastocystis*-positive IBS patients; Control/B-: *Blastocystis*-negative control subjects; Control/B + : *Blastocystis*-positive control subjects. **(C,D)** LefSe (LDA Effect Size) analysis in the control group **(C)** and the IBS group **(D)**. Red, taxa higher in *Blastocystis*-infected subjects; green, taxa higher in non-*Blastocystis*-infected subjects. Statistical significance was determined by LefSe analysis with FDR correction (only those species with *q* values < 0.05 and LDA effect size > 2 are shown). **(E,F)** Beta diversity analysis: PCoA of the unweighted UniFrac distance of non-carriers (yellow plots) and carriers (blue plots) of *Blastocystis* in the control group **(E)** and the IBS group **(B)**.

At the phylum level, the *Firmicutes/Bacteroidetes* ratio was decreased in the IBS-C group (Figure 2A). *Firmicutes* were significantly decreased in IBS-C subjects (Mann–Whitney test, *p* = 0.012), whereas *Proteobacteria* and *Verrucomicrobia* were significantly increased (Mann–Whitney test, *p* = 0.046 and 0.035, respectively). Moreover, β-diversity analyses confirmed differences between bacterial communities of IBS-C and controls at the OTU level (based on Bray–Curtis dissimilarities, Figure 2B). The *Firmicutes/Bacteroidetes* ratio tended to be increased in *Blastocystis* carriers from both IBS-C and control groups when compared to non-carrier groups (Mann–Whitney test, *p* = 0.085 and *p* = 0.449, respectively, Figure 3A). Interestingly, whereas no phylum was significantly different between *Blastocystis*-negative and -positive controls (Table 1), significant differences were observed within IBS subjects. *Tenericutes* phylum was significantly expanded among *Blastocystis* carriers of the IBS-C group (Figure 3B). In the *Blastocystis-*positive control group, a trend to an increase of *Tenericutes* was also observed (Figure 3B). Enrichment and depletion of several bacterial taxa were further confirmed by LEfSe (linear discriminant analysis effect size) analysis (*p* < 0.05, LDA > | 2|; Figures 3C,D and Supplementary Figure 1). Interestingly, *Ruminococcaceae* were enriched in the control group when subjects were positive for *Blastocystis* (Figure 3C). In IBS-C patients, *Lactobacillus* were decreased when subjects were colonized with *Blastocystis* (Figure 3D). The PCoA based on Bray–Curtis dissimilarities and unweighted or weighted Unifrac distances showed a modest clustering of the samples from IBS-C according to *Blastocystis* carriage, but not for controls (Figures 3E,F).

**TABLE 1:** Relative abundances of bacterial phyla.

	IBS group			Control group		
		
Phyla	*Blastocystis*	No *Blastocystis*	*p*-value	*Blastocystis*	No *Blastocystis*	*p*-value
*Actinobacteria*	0.006	0.004	0.548	0.002	0.006	0.090
*Bacteroidetes*	0.366	0.468	**0.046**	0.340	0.412	0.347
*Epsilonbacteraeota*	0.009	4.52 × 10^–5^	**<0.001**	7.40 × 10^–5^	5.29 × 10^–5^	0.740
*Firmicutes*	0.429	0.382	0.595	0.479	0.498	0.880
*Fusobacteria*	0.013	7.14 × 10^–5^	**0.018**	9.39 × 10^–5^	8.77 × 10^–5^	0.976
*Lentisphaerae*	0.002	0.003	0.771	0.005	0.004	0.525
*Proteobacteria*	0.094	0.105	0.986	0.039	0.055	0.525
*Spirochaetes*	0	0	/	2.24 × 10^–5^	0	0.148
*Synergistetes*	0.002	8.64 × 10^–6^	**0.037**	1.70 × 10^–5^	3.16 × 10^–4^	0.610
*Tenericutes*	0.036	0.013	**0.049**	0.108	0.008	0.059
*Verrucomicrobia*	0.040	0.005	**0.046**	0.002	0.001	0.786

*For statistical analysis, Mann–Whitney test was performed.*

*Significant *p*-values appeared in bold.*

**FIGURE 4:**
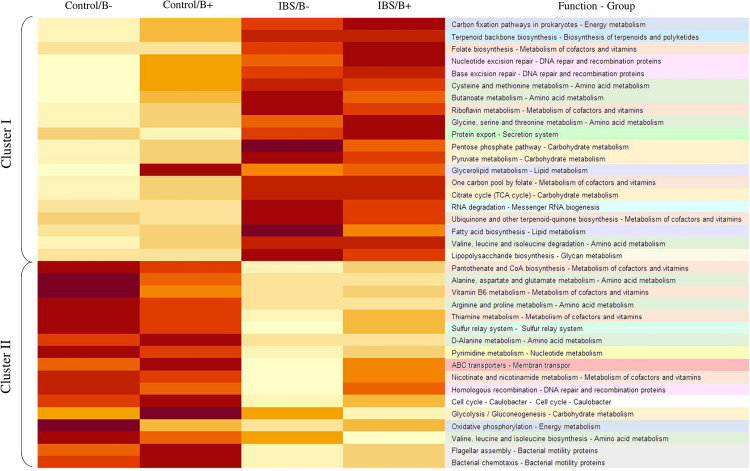
Heat map illustrating changes in functional capabilities in the four groups of patients. The color scale ranges from light yellow to dark red: light yellow indicates a low functional enrichment, and dark red indicates a high functional enrichment of the averaged individual KEGG function.

To further explore the impact of microbiota differences, a functional prediction of the microbial capabilities within each group was conducted *in silico*. An important difference was observed between IBS patients and healthy subjects, where two major categories of functional groups emerged. Cluster I fitted with a functional enrichment increase in IBS group, whereas in cluster II, there was a decrease in enrichment (Figure 4). Genes concerning metabolism of cofactors and vitamins, or amino acid metabolism were found in both clusters. There were a number of genes annotated to functions involved in the metabolism of complex carbohydrates, most of these are in Cluster I, but genes implicated in glycolysis/gluconeogenesis were identified in cluster II. Genes linked with lipid metabolism such as glycerolipid metabolism and fatty acid biosynthesis were only present in Cluster I. The only significant difference in functional capabilities according to the presence of *Blastocystis* in a same group (IBS vs. healthy) concerns glycerolipid metabolism among healthy subjects (1.027 for *Blastocystis* carriers vs. 0.964 for non-carriers, Student’s *t*-test *p* = 0.032). No difference was observed for this pathway in the IBS group according to carriage or not of *Blastocystis* (*p* = 0.919).

In the 18S rRNA sequence data set, 13 samples had fewer than 650 sequences per sample after applying filtering steps, which prompted their removal from the data set. Thus, 45 samples (25 IBS and 20 controls) were retained for ecological analysis of eukaryotes.

**FIGURE 5:**
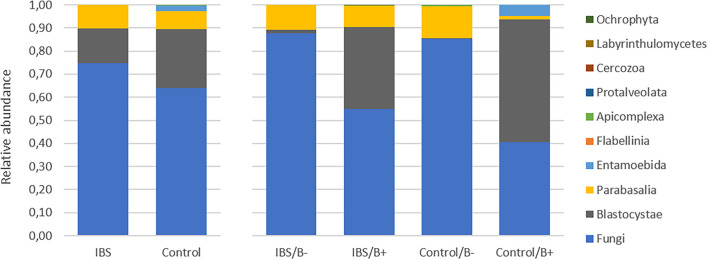
Comparison of the fecal eukaryotic microbiota composition between controls and IBS subjects at the class level. IBS/B-: *Blastocystis*-negative IBS patients; IBS/B + : *Blastocystis*-positive IBS patients; Control/B-: *Blastocystis*-negative control subjects; Control/B + : *Blastocystis*-positive control subjects.

**FIGURE 6:**
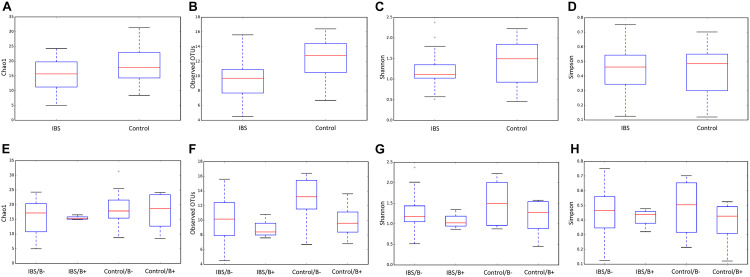
Comparison of alpha diversity indices of the fecal fungal microbiota between controls **(E–H)** and IBS-C subjects **(A–D)** according to *Blastocystis* carriage. **(A,E)** Chao 1, **(B,F)** observed number of OTUs, **(C,G)** Shannon diversity, and **(D,H)** Simpson’s index. Significant (or close to significance) *p*-values were reported on boxplots. IBS/B-: *Blastocystis*-negative IBS patients; IBS/B + : *Blastocystis*-positive IBS patients; Control/B-: *Blastocystis*-negative control subjects; Control/B + : *Blastocystis*-positive control subjects.

**FIGURE 7:**
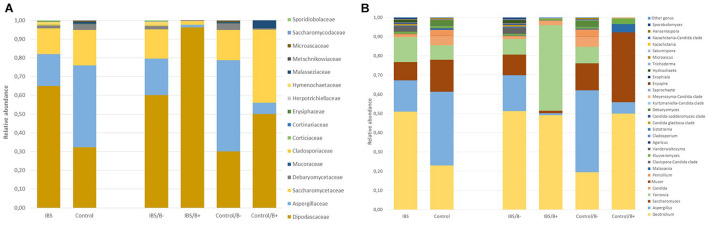
Comparison of the fecal mycobiota composition between controls and IBS subjects at family **(A)** and genus **(B)** levels. IBS/B-: *Blastocystis*-negative IBS patients; IBS/B+: *Blastocystis*-positive IBS patients; Control/B-: *Blastocystis*-negative control subjects; Control/B+: *Blastocystis*-positive control subjects.

As expected, the *Fungi* class accounted for the largest fraction of the eukaryotic microbiota in IBS-C and control groups (75 and 64%, respectively, Figure 5). *Blastocystis* was obviously the most abundant protist as we selected carriers for the study. Other protists identified in our study were *Dientamoeba fragilis* (*Parabasalia* class) and *Entamoeba* sp. (*Entamoeba* class). We further conducted an analysis of the *Fungi* class after filtering all the others eukaryota. Then, 14 more samples had fewer than 650 sequences per sample, which prompted their removal from the data set. Thus, 31 samples (17 IBS and 14 controls) were retained. Alpha diversity metrics were not significantly different between IBS and controls (Figures 6A–D). *Blastocystis* carriage had no impact on alpha diversity (Figures 6E–H). At the family level, *Dipodascaceae*, *Aspergillaceae*, and *Saccharomycetaceae* were the most abundant (64, 17, and 16% in the IBS group, and 31, 41, and 18% in controls, respectively; Figure 7A). Within the two groups of patients, *Dipodascaceae* were increased among *Blastocystis* carriers, while *Aspergillaceae* were decreased (Figure 7A; Table 2). *Saccharomycetaceae* were more than twofold higher in controls carrying *Blastocystis* compared to controls not colonized (39 and 15%, respectively). Considering the 10 most prevalent families, *Aspergillaceae* tended to be increased among controls not carrying *Blastocystis* compared to positive controls (Table 2). *Metschnikowiaceae* were increased among IBS subjects carrying *Blastocystis* compared to non-carriers (Table 2). *Geotrichum*, *Aspergillus*, *Saccharomyces*, and *Yarrowia* were the most abundant genera (51, 16, 9, and 13% in the IBS group, and 23, 38, 17, and 8% in controls, respectively; Figure 7B). *Clavispora* relative abundance was significantly decreased among IBS patients compared to healthy subjects (1.47 × 10^–5^ and 2.52 × 10^–3^, respectively, Mann–Whitney test, *p* = 0.031). In the same way, there was also a decrease close to significance of *Penicillium* relative abundance (6.04 × 10^–3^ and 3.09 × 10^–2^, respectively, Mann–Whitney test, *p* = 0.063). The relative abundance and *p*-values of the most abundant genera in our study and of the genera of the core mycobiota are reported in Table 2. Considering the relative abundance, *Penicillium* were significantly reduced in *Blastocystis* carriers of the control group. *Clavispora* and *Trichosporon* were significantly increased in *Blastocystis* carriers of the IBS group (Table 2).

**TABLE 2:** Relative abundances of the 10 most prevalent fungal families and the most commonly detected genera in gut mycobiome studies (core mycobiota) and in this study.

	IBS group			Control group		
		
	*Blastocystis*	No *Blastocystis*	*p*-value	*Blastocystis*	No *Blastocystis*	*p*-value
Families						
*Aspergillaceae*	0.013	0.193	0.362	0.060	0.460	0.056
*Cladosporiaceae*	0	0.003	0.574	0	3.553 × 10^–3^	0.184
*Corticiaceae*	0	0.002	0.758	0	0	/
*Debaryomycetaceae*	0.001	0.017	1.000	0.005	0.032	0.406
*Dipodascaceae*	0.937	0.594	0.231	0.499	0.283	0.454
*Herpotrichiellaceae*	0	0	/	0	0.001	0.184
*Hymenochaetaceae*	0	0.016	0.574	0	0	/
*Malasseziaceae*	0.001	0.001	0.441	0.043	0.003	0.944
*Metschnikowiaceae*	1.090 × 10^–4^	0	**0.045**	0	0.003	0.117
*Saccharomycetaceae*	0.021	0.153	0.592	0.391	0.152	0.945
Genera						
Aspergillus*	0.010	0.187	0.449	0.059	0.425	0.106
Candida*	0.021	0.009	0.945	3.09 × 10^–4^	0.054	0.096
Cladosporium*	0	0.003	0.574	0	3.55 × 10^–4^	0.184
*Clavispora*	1.09 × 10^–4^	0	**0.045**	0	0.003	0.117
Debaryomyces*	0	6.78 × 10^–5^	0.351	0	0.031	0.279
*Geotrichum*	0.491	0.512	0.281	0.499	0.195	0.635
*Kluyveromyces*	0.008	0.010	0.941	0.029	0.010	1
Malassezia*	7.65 × 10^–4^	0.001	0.441	0.043	0.003	0.943
Penicillium*	0.003	0.006	1	3.86 × 10^–4^	0.035	**0.006**
Saccharomyces*	0.013	0.107	0.571	0.361	0.141	0.944
Trichosporon*	0.003	0	**0.045**	0	9.08 × 10^–4^	0.279
*Vanderwaltozyma*	0	0.033	0.274	3.09 × 10^–4^	6.81 × 10^–4^	1
*Yarrowia*	0.445	0.081	0.639	2.32 × 10^–4^	0.085	0.184

For statistical analysis, Mann–Whitney test was performed. *: genera belonging to the core mycobiota.

*Significant *p*-values appeared in bold.*

## Discussion

Our study was motivated by the literature of the last decade reporting high prevalence of *Blastocystis* in IBS patients, the concerns about the risk associated with *Blastocystis* in fecal microbiota transplantation, and recent studies in animal models (Nourrisson et al., 2014; Terveer et al., 2019; Defaye et al., 2020). Recent works have studied the impact of *Blastocystis* in IBS-D patients, but none in IBS-C (Nagel et al., 2016). As each type of IBS is considered to be profoundly different from each other, we decided to focus on IBS-C patients. So, our data are the first to describe both prokaryotic and eukaryotic microbiota in IBS-C patients colonized with *Blastocystis*.

Classical IBS-C symptoms were described by patients, mostly female as expected with IBS, whether or not they were carriers of *Blastocystis*. Particularly, severity, based on Francis score, was not different according to *Blastocystis* carriage. All patients consume conventional treatments (antispasmodic, laxatives, transit accelerators, anti-bloating, etc.), periodically or over the long term, so it was difficult to compare them with each other. Taking probiotics was a non-inclusion criterion in order not to induce bias during the study of the microbiota. Thus, patients enrolled in this study are permitted to constitute comparable groups.

As previously reported, we observed a decrease in the prokaryotic microbiota richness of IBS-C subjects. Decrease in alpha diversity was reported from numerous studies interested in chronic intestinal or extra-intestinal diseases and is suspected to be associated with an altered function of gut microbiota (Le Chatelier et al., 2013). Conversely, a higher bacterial diversity is commonly associated with good health and lower incidence of inflammatory diseases (Loh and Blaut, 2012). *Blastocystis* was reported to be strongly associated with broad shifts in the gut-resident bacterial community and an increase in bacterial alpha diversity (Deng et al., 2021). In our study, we did not find significant increase of alpha diversity in *Blastocystis* carriers from the control group, but a trend was observed in IBS-C. Facing this increase in alpha diversity associated with *Blastocystis* carriage, some authors suggested that *Blastocystis* may be a component of a healthy microbiota (Deng et al., 2021). This point remains to be clarified as *Blastocystis* is reported to be more frequent in IBS patients, which present a dysbiosis. Moreover, pathogenic protozoan, such as *Giardia duodenalis*, *Entamoeba histolytica*, or *Cryptosporidium* sp., were associated with an increase in gut bacterial diversity or with altered microbiome profiles compared to uninfected people (Burgess and Petri, 2016; Fekete et al., 2020).

At the taxonomic level, our results were congruent with the literature, as we observed in IBS-C patients an increase in the *Bacteroidetes* phylum leading to an inversion of the *Firmicutes*/*Bacteroidetes* ratio, and an increase in the *Proteobacteria* phylum (Pittayanon et al., 2019). The *Firmicutes/Bacteroidetes* ratio was increased in *Blastocystis* carriers of both IBS-C and control groups, which is in favor of a less inflammatory gut environment. In our study, *Blastocystis* carriage was associated with a high relative abundance of the *Tenericutes* phylum, which is composed of commensals and pathogens (Wang et al., 2020). Even though this phylum is frequently modified in microbiota studies, little is known about its involvement in human health. In control group, we also observed in *Blastocystis*-positive individuals an increase in *Ruminococcaceae*, which are key symbionts of the gut ecosystem. This result was previously observed in subjects infected with *E. histolytica* or *E. dispar* (Morton et al., 2015). Interestingly, we recently described in rat an increase of *Tenericutes* phylum after experimental infections with *Blastocystis*, suggesting that this phylum may be specifically modified by the parasite, independently of the underlying intestinal disease (Defaye et al., 2020).

Functional predictions of the microbial capabilities were strongly impacted by the IBS pathology, irrespective of *Blastocystis* status. Due to this significant impact of IBS, it did not seem relevant to us to look for the differences in functional capacities by taking into account only the presence of *Blastocystis*. KEGG orthology pathway of glycerolipid metabolism was found to be significantly enriched in healthy subjects carrying *Blastocystis* compared to non-carrier healthy subjects. The role of the parasite on enrichment of genes involved in this metabolism has to be confirmed with further studies.

In the gut ecosystem, fungi and bacteria directly interact with each other (Lynch and Pedersen, 2016). Despite being a minor component of the gut microbial community, evidences indicated that fungi can play a role in gut diseases, as demonstrated in inflammatory bowel disease (Sokol et al., 2017). Whereas extensive literature is available about prokaryotic dysbiosis associated with IBS, few studies were interested in eukaryoma. Fungal dysbiosis has been reported in IBS with an enrichment of *Saccharomycetes* and a decrease of alpha diversity (Botschuijver et al., 2017; Sciavilla et al., 2021). Considering the fecal eukaryoma, and more precisely the *Fungi* class, the relative abundance of *Dipodascaceae* was twofold higher in IBS-C subjects compared to controls. *Clavispora* and *Penicillium* genera were decreased in IBS-C compared to healthy subjects.

With the work by Nieves- Ramirez, our study is the second to address intestinal eukaryotic microflora associated with *Blastocystis* carriage. In our study, *Blastocystis* colonization was associated with more discrete differences in the eukaryotic microbiota compared to prokaryotic shifts, without modification of alpha or beta diversity. Nieves-Ramirez and colleagues reported recently a modification of fungal microbiota of a rural Mexican population colonized with *Blastocystis* (Nieves-Ramírez et al., 2018). They reported an increase in yeast and fungal species (*Debaryomyces hansenii, Mucor mucedo, Aspergillus flavus, Mucor racemosus*, and *Issatchenkia terricola*) and a decrease of *Hymenolepis nana* in patients colonized with *Blastocystis*. The transposition of these results is not possible to a population of industrialized countries, where infections due to *H. nana* are absent. We did not observe significant modifications of *Debaryomyces* and *Mucor* genera according to *Blastocystis* carriage. These differences could be explained by a different region of the 18S rRNA gene targeted in the two studies. Diet could also have impacted proportions of some fungi (Richard and Sokol, 2019). Interestingly, in our results, *Dipodascaceae* family was increased in the presence of the parasite in the two groups of patients, whereas *Aspergillaceae, Aspergillus*, and *Penicillium* were decreased. At the opposite end, the modifications of some groups were different depending on whether one considered the IBS or the control groups. *Clavispora* were significantly increased in IBS patients carrying the parasite, whereas in the control group, this genus was only encountered in negative subjects. *Clavispora* has been reported to be increased in patients suffering from Crohn’s disease (Lewis et al., 2015). Interestingly, when *Blastocystis* was present in the IBS group, the modifications were the same as previously described in Dectin-1 knockout mice; i.e., the proportion of opportunistic pathogenic fungi including *Candida* and *Trichosporon* increased, whereas non-pathogenic *Saccharomyces* decreased (Iliev et al., 2012). These mice experienced more severe DSS-induced colitis than wild-type mice, suggesting that this mycobiota pattern could be linked to inflammatory environment in predisposed animals. These modifications were not observed in healthy subjects in whom *Saccharomyces* genus was increased when *Blastocystis* was present. *Saccharomyces* are known to limit inflammatory response and increase immune health; these yeasts have also been used as probiotics (Kourelis et al., 2010). No differences were observed among the other genera that may constitute the core mycobiota (i.e., gut residents) previously described (Richard and Sokol, 2019).

We recognize that our study presents some limitations. First, a limited number of patients were included. This impacts on the analysis of secondary criteria. Also, we did not address the link between *Blastocystis* STs and microbiota modifications because we were limited by the number of subjects included. The role of *Blastocystis* ST was mentioned several times, but this hypothesis needs further investigations (Yason et al., 2019; Deng et al., 2021). Francis score subgroup analysis could not be performed either. However, it would be interesting to investigate a correlation between changes in the microbiota of patients and the severity of their symptoms. Moreover, the presence of other parasites may also have slightly affected our results. Indeed, two patients of the control group were positive for *Entamoeba* sp. and a low rate of *Blastocystis*/*D. fragilis* co-carriages was detected in both IBS-C and control groups. Nevertheless, it underlies the difficulty to interpret to what degree *Blastocystis* contributes to microbiota changes in human studies. So, our results suggest that when studying the link between *Blastocystis* (or other parasites) and prokaryotic microbiota in human, we should also perform analyses of associated eukaryoma.

To conclude, *Blastocystis* is associated with modifications of the fecal microbiota, but which do not go in the same direction depending on whether we consider healthy people or IBS-C patients, particularly for the mycobiota. Even though animal models have highlighted some modifications specifically attributable to this parasite, we still do not know for most of the microbiota changes if they are due to the presence of *Blastocystis*. So, further experimentations with animal models using various *Blastocystis* ST, and/or humanized microbiota models, could help to understand the role of *Blastocystis* in the gut environment.

## Data Availability Statement

The datasets generated for this study can be found in NCBI PRJNA730687.

## Ethics Statement

The studies involving human participants were reviewed and approved by this clinical study was approved by the Research Ethics Committees of the Hospital of Clermont-Ferrand (“Comité de Protection des Personnes Sud-Est VI,” France) with the reference number 2013-A00031-44. The patients/participants provided their written informed consent to participate in this study.

## Author Contributions

CN, MD, and PP: conceptualization. CN, JS, and PP: methodology. CN and PP: software, formal analysis, and visualization. CN, FD, MD, and PP: validation. CN, JS, JB, and MD: investigation. FD and PP: resources. CN: data curation and writing – original draft preparation. JS, JB, FD, MD, and PP: writing, review, and editing. FD, MD, and PP: supervision and funding acquisition. All authors contributed to the article and approved the submitted version.

## Conflict of Interest

The authors declare that the research was conducted in the absence of any commercial or financial relationships that could be construed as a potential conflict of interest.

## Publisher’s Note

All claims expressed in this article are solely those of the authors and do not necessarily represent those of their affiliated organizations, or those of the publisher, the editors and the reviewers. Any product that may be evaluated in this article, or claim that may be made by its manufacturer, is not guaranteed or endorsed by the publisher.

## Footnotes

1
www.mrdnalab.com

2
http://qiime.org/

3
https://www.arb-silva.de

4
http://tax4fun.gobics.de

## Supplementary Material

Supplementary Figure 1 LDA effect size, log_10_ transformed *q* value (FDR-adjusted *p* value) and species annotation are shown. **(A)** Control group, **(B)** IBS group. Green bars indicate species enriched in non-infected subjects, while red bars indicate species enriched in *Blastocystis*-infected subjects. Statistical significance was determined by LefSe analysis with FDR correction (only those species with *q* values < 0.05 and LDA effect size > 2 are shown).

Supplementary Table 1 Sequences of the primers used in the study.

Supplementary Table 2 Demographic characteristics and clinical data of patients included. F, female; M, male; nd, not determined; na, not applicable.

## References

[B1] Alfellani M. A. Taner-Mulla D. Jacob A. S. Imeede C. A. Yoshikawa H. Stensvold C. R. (2013). Genetic diversity of blastocystis in livestock and zoo animals. *Protist* 164 497–509. 10.1016/j.protis.2013.05.003 23770574

[B2] Aßhauer K. P. Wemheuer B. Daniel R. Meinicke P. (2015). Tax4Fun: predicting functional profiles from metagenomic 16S rRNA data. *Bioinformatics* 31 2882–2884. 10.1093/bioinformatics/btv287 25957349 PMC4547618

[B3] Botschuijver S. Roeselers G. Levin E. Jonkers D. M. Welting O. Heinsbroek S. E. M. (2017). Intestinal fungal dysbiosis associates with visceral hypersensitivity in patients with irritable bowel syndrome and rats. *Gastroenterology* 153 1026–1039. 10.1053/j.gastro.2017.06.004 28624575

[B4] Burgess S. L. Petri W. A. (2016). The intestinal bacterial microbiome and e. histolytica infection. *Curr. Trop. Med. Rep.* 3 71–74. 10.1007/s40475-016-0083-1 27525214 PMC4967426

[B5] Cammarota G. Ianiro G. Tilg H. Rajilić-Stojanović M. Kump P. Satokari R. (2017). European consensus conference on faecal microbiota transplantation in clinical practice. *Gut* 66 569–580. 10.1136/gutjnl-2016-313017 28087657 PMC5529972

[B6] Caporaso J. G. Kuczynski J. Stombaugh J. Bittinger K. Bushman F. D. Costello E. K. (2010). QIIME allows analysis of high-throughput community sequencing data. *Nat. Methods* 7 335–336. 10.1038/nmeth.f.303 20383131 PMC3156573

[B7] Defaye M. Nourrisson C. Baudu E. Lashermes A. Meynier M. Meleine M. (2020). Fecal dysbiosis associated with colonic hypersensitivity and behavioral alterations in chronically blastocystis-infected rats. *Sci. Rep.* 10:9146. 10.1038/s41598-020-66156-w 32499543 PMC7272397

[B8] Deng L. Wojciech L. Gascoigne N. R. J. Peng G. Tan K. S. W. (2021). New insights into the interactions between blastocystis, the gut microbiota, and host immunity. *PLoS Pathog.* 17:e1009253. 10.1371/journal.ppat.1009253 33630979 PMC7906322

[B9] El Safadi D. Cian A. Nourrisson C. Pereira B. Morelle C. Bastien P. (2016). Prevalence, risk factors for infection and subtype distribution of the intestinal parasite blastocystis sp. from a large-scale multi-center study in France. *BMC Infect. Dis.* 16:451. 10.1186/s12879-016-1776-8 27566417 PMC5002209

[B10] Fekete E. Allain T. Siddiq A. Sosnowski O. Buret A. G. (2020). Giardia spp. and the gut microbiota: dangerous liaisons. *Front. Microbiol.* 11:618106. 10.3389/fmicb.2020.618106 33510729 PMC7835142

[B11] Iliev I. D. Funari V. A. Taylor K. D. Nguyen Q. Reyes C. N. Strom S. P. (2012). Interactions between commensal fungi and the C-Type lectin receptor dectin-1 influence colitis. *Science* 336 1314–1317. 10.1126/science.1221789 22674328 PMC3432565

[B12] Kourelis A. Kotzamanidis C. Litopoulou-Tzanetaki E. Papaconstantinou J. Tzanetakis N. Yiangou M. (2010). Immunostimulatory activity of potential probiotic yeast strains in the dorsal air pouch system and the gut mucosa. *J. Appl. Microbiol.* 109 260–271. 10.1111/j.1365-2672.2009.04651.x 20059615

[B13] Le Chatelier E. Nielsen T. Qin J. Prifti E. Hildebrand F. Falony G. (2013). Richness of human gut microbiome correlates with metabolic markers. *Nature* 500 541–546. 10.1038/nature12506 23985870

[B14] Lewis J. D. Chen E. Z. Baldassano R. N. Otley A. R. Griffiths A. M. Lee D. (2015). Inflammation, antibiotics, and diet as environmental stressors of the gut microbiome in pediatric Crohn’s disease. *Cell Host Microbe* 18 489–500. 10.1016/j.chom.2015.09.008 26468751 PMC4633303

[B15] Li H. Qu J. Li T. Li J. Lin Q. Li X. (2016). Pika population density is associated with the composition and diversity of gut microbiota. *Front. Microbiol.* 7:758. 10.3389/fmicb.2016.00758 27242770 PMC4870984

[B16] Loh G. Blaut M. (2012). Role of commensal gut bacteria in inflammatory bowel diseases. *Gut Microbes* 3 544–555. 10.4161/gmic.22156 23060017 PMC3495792

[B17] Longstreth G. F. Thompson W. G. Chey W. D. Houghton L. A. Mearin F. Spiller R. C. (2006). Functional bowel disorders. *Gastroenterology* 130 1480–1491. 10.1053/j.gastro.2005.11.061 16678561

[B18] Lynch S. V. Pedersen O. (2016). The human intestinal microbiome in health and disease. *N. Engl. J. Med.* 375 2369–2379. 10.1056/NEJMra1600266 27974040

[B19] Morton E. R. Lynch J. Froment A. Lafosse S. Heyer E. Przeworski M. (2015). Variation in rural african gut microbiota is strongly correlated with colonization by entamoeba and subsistence. *PLoS Genet.* 11:e1005658. 10.1371/journal.pgen.1005658 26619199 PMC4664238

[B20] Nagel R. Traub R. J. Allcock R. J. N. Kwan M. M. S. Bielefeldt-Ohmann H. (2016). Comparison of faecal microbiota in blastocystis-positive and blastocystis-negative irritable bowel syndrome patients. *Microbiome* 4:47. 10.1186/s40168-016-0191-0 27580855 PMC5007835

[B21] Nieves-Ramírez M. E. Partida-Rodríguez O. Laforest-Lapointe I. Reynolds L. A. Brown E. M. Valdez-Salazar A. (2018). Asymptomatic intestinal colonization with protist blastocystis is strongly associated with distinct microbiome ecological patterns. *Msystems* 3:e00007. 10.1128/mSystems.00007-18 29963639 PMC6020473

[B22] Nourrisson C. Scanzi J. Pereira B. NkoudMongo C. Wawrzyniak I. Cian A. (2014). Blastocystis is associated with decrease of fecal microbiota protective bacteria: comparative analysis between patients with irritable bowel syndrome and control subjects. *PloS One* 9:e111868. 10.1371/journal.pone.0111868 25365580 PMC4218853

[B23] Nourrisson C. Wawrzyniak I. Cian A. Livrelli V. Viscogliosi E. Delbac F. (2016). On blastocystis secreted cysteine proteases: a legumain-activated cathepsin b increases paracellular permeability of intestinal caco-2 cell monolayers. *Parasitology* 143 1713–1722. 10.1017/S0031182016001396 27609526

[B24] Parfrey L. Wegener W. A. Walters C. L. Lauber J. C. Clemente D. Berg-Lyons C. (2014). Communities of microbial eukaryotes in the mammalian gut within the context of environmental eukaryotic diversity. *Front. Microbiol.* 5:298. 10.3389/fmicb.2014.00298 24995004 PMC4063188

[B25] Pittayanon R. Lau J. T. Yuan Y. Leontiadis G. I. Tse F. Surette M. (2019). Gut microbiota in patients with irritable bowel syndrome-a systematic review. *Gastroenterology* 157 97–108. 10.1053/j.gastro.2019.03.049 30940523

[B26] Poirier P. Wawrzyniak I. Albert A. El Alaoui H. Delbac F. Livrelli V. (2011). Development and evaluation of a real-time PCR assay for detection and quantification of blastocystis parasites in human stool samples: prospective study of patients with hematological malignancies. *J. Clin. Microbiol.* 49 975–983. 10.1128/JCM.01392-10 21177897 PMC3067686

[B27] Poirier P. Wawrzyniak I. Vivarès C. P. Delbac F. El Alaoui H. (2012). New insights into *Blastocystis spp*.: a potential link with irritable bowel syndrome. *PLoS Pathog.* 8:e1002545. 10.1371/journal.ppat.1002545 22438803 PMC3305450

[B28] Ramírez J. D. Sánchez A. Hernández C. Flórez C. Consuelo Bernal M. Giraldo J. C. (2016). Geographic distribution of human blastocystis subtypes in South America. *Infect. Genet. Evol.* 41 32–35. 10.1016/j.meegid.2016.03.017 27034056

[B29] Richard M. L. Sokol H. (2019). The gut mycobiota: insights into analysis, environmental interactions and role in gastrointestinal diseases. *Nat. Rev. Gastroenterol. Hepatol.* 16 331–345. 10.1038/s41575-019-0121-2 30824884

[B30] Sciavilla P. Strati F. Di Paola M. Modesto M. Vitali F. Cavalieri D. (2021). Gut microbiota profiles and characterization of cultivable fungal isolates in IBS patients. *Appl. Microbiol. Biotechnol.* 105 3277–3288. 10.1007/s00253-021-11264-4 33839797 PMC8053167

[B31] Segata N. Izard J. Waldron L. Gevers D. Miropolsky L. Garrett W. S. (2011). Metagenomic biomarker discovery and explanation. *Genome Biol.* 12:R60. 10.1186/gb-2011-12-6-r60 21702898 PMC3218848

[B32] Sokol H. Leducq V. Aschard H. Pham H.-P. Jegou S. Landman C. (2017). Fungal microbiota dysbiosis in IBD. *Gut* 66 1039–1048. 10.1136/gutjnl-2015-310746 26843508 PMC5532459

[B33] Stensvold C. R. Clark C. G. (2020). Pre-empting pandora’s box: blastocystis subtypes revisited. *Trends Parasitol.* 36 229–232. 10.1016/j.pt.2019.12.009 32001133

[B34] Terveer E. M. van Gool T. Ooijevaar R. E. I Sanders M. J. G. Boeije-Koppenol E. Keller J. J. (2019). Human transmission of blastocystis by fecal microbiota transplantation without development of gastrointestinal symptoms in recipients. *Clin. Infect. Dis.* 71 2630–2636. 10.1093/cid/ciz1122 31728525 PMC7745006

[B35] Wang Y. Huang J.-M. Zhou Y.-L. Almeida A. Finn R. D. Danchin A. (2020). Phylogenomics of expanding uncultured environmental tenericutes provides insights into their pathogenicity and evolutionary relationship with bacilli. *BMC Genomics* 21:408. 10.1186/s12864-020-06807-4 32552739 PMC7301438

[B36] Yason J. A. Liang Y. R. Png C. W. Zhang Y. Tan K. S. W. (2019). Interactions between a pathogenic blastocystis subtype and gut microbiota: in vitro and in vivo studies. *Microbiome* 7:30. 10.1186/s40168-019-0644-3 30853028 PMC6410515

