# Supplementary figures and images for: Prokaryotic and Eukaryotic Fecal Microbiota in Irritable Bowel Syndrome Patients and Healthy Individuals Colonized With Blastocystis
Source: Front Microbiol. 2021 Sep 17;12:713347. doi: 10.3389/fmicb.2021.713347 (PMC8486285; doi:10.3389/fmicb.2021.713347)

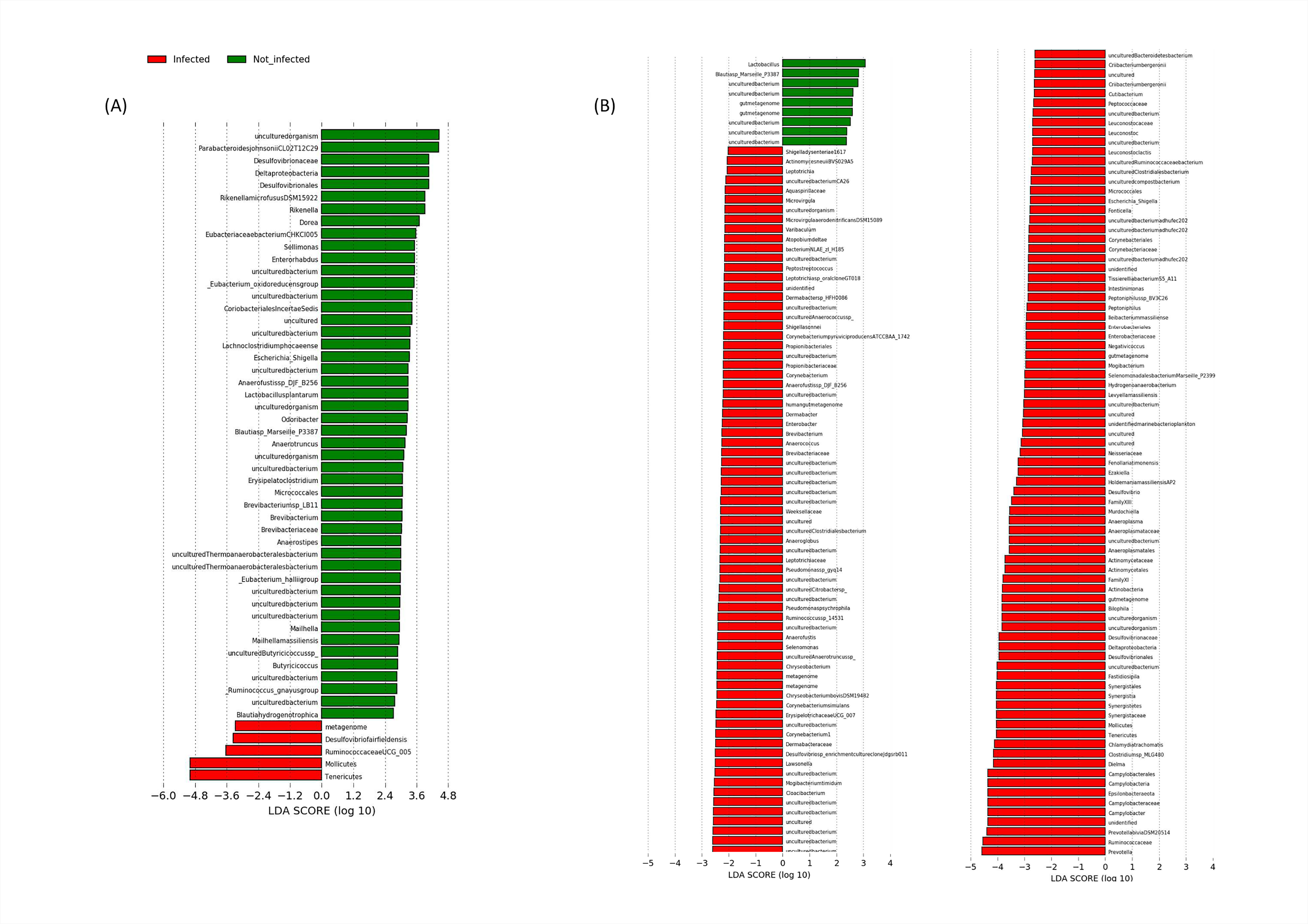

Supplement: Supplementary Figure 1 — LDA effect size, log10 transformed q value (FDR-adjusted p value) and species annotation are shown. (A) Control group, (B) IBS group. Green bars indicate species enriched in non-infected subjects, while red bars indicate species enriched in Blastocystis-infected subjects. Statistical significance was determined by LefSe analysis with FDR correction (only those species with q values < 0.05 and LDA effect size > 2 are shown). [file Image_1.TIFF]
