# Supplementary material for: Prokaryotic and Eukaryotic Fecal Microbiota in Irritable Bowel Syndrome Patients and Healthy Individuals Colonized With Blastocystis
Source: Front Microbiol. 2021 Sep 17;12:713347. doi: 10.3389/fmicb.2021.713347 (PMC8486285; doi:10.3389/fmicb.2021.713347)
Supplement: Supplementary Table 1 — Sequences of the primers used in the study. [file Table_1.DOCX]

| **Target** | **Name** | **Sequence 5’-3’** | **Amplicon size (bp)** | **References** |
| --- | --- | --- | --- | --- |
| *Blastocystis* spp. (SSU rRNA) | BL18SPPF1  BL18SR2PP | AGT AGT CAT ACG CTC GTC TCA AA  TCT TCG TTA CCC GTT ACT GC | 320 to 342 | Poirier et al. 2011 |
| 16S rRNA (V3-V5) | 515F  909R | GTG YCA GCM GCC GCG GTA  CCC CGY CAA TTC MTT TRA GT | 459 | Li et al. 2016 |
| 18S rRNA | 515F_Euk  1119R | GTG CCA GCM GCC GCG GTA A  GGT GCC CTT CCG TCA | 600 | Parfrey et al. 2014 |
